# William Jackson Schull and mutation studies on human cohorts

**DOI:** 10.3389/fpubh.2023.1151861

**Published:** 2023-03-17

**Authors:** Tomoko Y. Steen

**Affiliations:** Department of Microbiology and Immunology, Georgetown University School of Medicine, Washington, DC, United States

**Keywords:** mutation, human genetics, ionizing radiation, ABCC, RERF, children of atomic bomb, biological effect of radiation

## 1. Introduction

In March 1993, “If you are interested in radiation-induced mutations on humans, you must meet Jack Schull!” said James Crow^1^in our casual conversation at the National Institute of Genetics (NIG) in Mishima, Japan. This was the first time I encountered the name William Jackson Schull. I was conducting my Ph.D. dissertation on the Nearly Neutral theory of molecular evolution developed by Dr. Tomoko Ohta (NIG) and learning about her biographical background (1). I spent a year at NIG, from August 1992 to August 1993. Mutation mechanisms and the generational effects of radiation have been among my lifetime interests as someone who grew up in Nagasaki as a second-generation atomic bomb survivor. I also met survivors as patients when I practiced as a pharmacist at a local general hospital in Nagasaki.

William Jackson Schull (17 March 1922−20 June 2017) was a geneticist who contributed his life's work to studies of the biological effects of ionizing radiation on human cohorts. His Ph.D. dissertation focused on the genetic inheritance of specific characters and mutations (2). In his interview with me, Schull said his choice to take the lead at the Atomic Bomb Casualty Commission (ABCC) in Hiroshima and Nagasaki rather than taking a professorship at McGill University changed the course of his life completely. While an internationally recognized research scientist, his kind and personable nature allowed him to develop close friendships with Japanese people he met in Hiroshima and Nagasaki. Later in his life, he wrote a personal account of the people in Hiroshima and Nagasaki in *Song Among the Ruins* (3). This book shows his affinity toward people in those towns. He often told me that he spent more time in Japan than in the US and considered Japan his second home.

Schull was also very active in establishing human genetics research infrastructure in the US. He was one of the founding members of the Department of Human Genetics at the University of Michigan. He was also the founding director of the Center for Demographic and Population Genetics (CDPG) at the University of Texas at Houston. His overall scientific contributions, in addition to studies on the effects of ionizing radiation on human health, were the role of heredity and the interaction of heredity and environment in the etiology of chronic disease, the effects of inbreeding in human populations, the mechanisms of adaptations to hypoxic conditions, and the genetic epidemiology of populations burdened by chronic diseases associated with low socio-economic status. He was also an excellent mentor to many young researchers. Schull was a prolific author and scientist, publishing 425 academic papers and 15 books. However, from my observation over the last 30 years, his work centered on mutation studies in human cohorts under ionizing radiation. When the Fukushima nuclear accident was reported, he immediately contacted me and asked if he could help those people.

## 2. Mutation studies

In 1901, a Dutch botanist and one of the first geneticists, Hugo de Vries (February 16, 1848—May 21, 1935), coined the term “mutation” when he observed a new form of evening primrose, *Oenothera lamarckiana, via* his plant crossing experiment (5). Mutationism (de Vries called *mutationstheorie*) alongside Mendelism, supported by geneticists such as William Bateson, Thomas Hunt Morgan, and Reginald Punnett, became widely accepted but initially seemed incompatible with Darwinism. This debate was resolved through the evolutionary synthesis in the 1930s (6). However, the discussion on the role of mutation evolved into another controversy of classical vs. balance theory in the mid-1900s. Masatoshi Nei, the author of *Mutation-Driven Evolution*, describes, “In the 1950s, population geneticists were divided into two camps, one camp supporting the “classical” theory and the other the “balance” theory (7). The “classical” theory asserted that most genetic variation within species is maintained by mutation-selection balance, whereas the “balance” theory proposed that genetic variation is maintained primarily by overdominant selection or some other type of balancing selection” (8).

One of the critical scientists of mutation studies who used radiation as a mutation inducer was Hermann Joseph Muller (December 21, 1890–April 5, 1967), who supported the “classical theory.” He received the Nobel Prize in Physiology or Medicine in 1946 “for discovering the production of mutations using X-ray irradiation” (The Nobel Prize in Physiology or Medicine 1946—NobelPrize.org). Due to his findings, Muller was concerned about the effects of radiation causing unwanted mutations among given species, including humans (9, 10). He warned of long-term dangers due to radioactive fallout from nuclear war and testing, and the threat of global public health issues, becoming a staunch anti-nuclear activist toward the end of his life (11–15).

Since the first half of the twentieth century, mutation studies have been the focus of geneticists, and radiation was one of the critical inducers for mutations. Various species were used for mutation studies, including fruit flies, mice, and plants. However, comprehensive data on human cohorts would eventually need to be analyzed to set a safety standard. The trends changed at the end of WWII.

## 3. ABCC/RERF findings (https://www.rerf.or.jp/en/)

The contribution of ABCC/RERF in the studies of the biological effects of radiation is indispensable, although controversial. Schull says in his review article published in 2003, “The children of atomic bomb survivors: a synopsis” following (16):

“In the autumn of 1945, when studies began to evaluate the physical and biological damage from the atomic bombing of Hiroshima and Nagasaki, public concern focused more on the genetic consequences than any other untoward health outcome. A wealth of experimental evidence existed attesting to the mutagenic effects of ionizing radiation on such diverse plant and animal species as *Drosophila melanogaster* (the fruit fly), *Zea mays* (corn), and even *Habrobracon juglandis* (the solitary wasp). This evidence suggested that the increase in mutations was linearly related to dose, implying that any dose, however small, would have genetic consequences. While it was assumed that the same must hold for the human species, it was the nature of these consequences and not the linearity with a dose that disturbed the public” (16).

Indeed, Atomic survivors' studies conducted by Schull and colleagues were the first studies of human populations exposed to a high radiation dose. The cohorts were significantly larger than those with radiation exposure through medical examinations and cancer treatments. Subsequently, studies have been conducted in a variety of cases, including residents in New Mexico after the Trinity test, military personnel after the nuclear testing in the pacific (17), clean-up workers and residents in Chornobyl (18), and most recently, people in Fukushima, Japan compared to other incidents (19).

Schull and his former Ph.D. advisor, James Neel's primary focus was radiation effects on fetuses and genetics at ABCC. One of the first noteworthy reports they published was on *The Effect of Exposure to the Atomic Bombs on Pregnancy Termination in Hiroshima and Nagasaki*, covering statistical methods, sex ratios, malformation data, and future studies (20). Schull's fundamental interest was mutation mechanisms in general, and he held many symposia to discuss such mutations. In 1962, he published an edited volume, *Mutation: The* Second *Conference on Genetics* (21). The conference was funded by The Josiah Marcy, Jr. Foundation and held on October 16–19, 1960, in Princeton, New Jersey. The three critical discussions for this conference were: “Problems of measurement of mutation rates,” “Mutagenesis, with particular reference to chemical factors,” and “Mutagens currently of potential significance to man and other species” (21).

Most of Schull's research projects on the biological effects of radiation for survivors of atomic bombs were supported and funded by the National Academy of Science (NAS)/National Research Council (NRC), Atomic Energy Commission (AEC), Department of Energy (DOE), and Japanese Ministry of Health and Welfare. In the preface of the report published under the National Academy of Sciences in 1991, *The Children of Atomic Survivors: A genetic study*, Dr. Frank Press, then President of the National Academy of Science, states that NAS and other agencies have “been engaged for some 45 years in an attempt to understand the late health effects on the survivors of the atomic bombings of Hiroshima and Nagasaki.” “One of the major questions demanding consideration has been the possible genetic effects of this exposure.” He believes the data “has also been of international importance in establishing standards for the protection of people in the workplace and the general public.” In this over 500-page report, Neel and Schull describe various aspects of genetic studies on atomic bomb survivors and their children (22). Schull knew that the Japanese practiced cousin marriages; thus, studies were also calibrated for related parents. Schull studied Japanese cousin marriage thoroughly, publishing a series of papers and even a book on the topic (23). The study of survivors, “Life Span Studies,” continues; however, this 1991 report covers wide-ranging questions and the need for a superior foundation of genetic studies of radiation exposure.

The most comprehensive report of the overall effects of atomic radiation for a half-century of studies conducted on survivors at ABCC and RERF is published in *Effects of Atomic Radiation*. The volume covers genetic effects, cancer, and other diseases caused by ionizing radiation (24). There was a discussion of publishing the further report in 2010.

## 4. Future studies: How do we explain the discrepancies between non-human species and human cohorts on outcomes of ionizing radiation?

The fundamental questions remaining for human cohorts on genetic and biological effects of ionizing radiation are (1) some discrepancies between non-human species and available human data on the effects of ionizing radiation, and (2) controversy of the low-dose radiation effects, considering whether the Linear no-threshold model (LNT) stands or not. One of my last conversations with Schull concerned people in Fukushima after the nuclear accident. While he devoted his life's work to the biological effects of radiation and discovered a massive amount of information, he expressed his frustration in not being able to help these nuclear accident survivors further by providing reliable information about the exposure to ionizing radiation at lower doses and what to expect due to of the discrepancies that exist between animal and human studies for generation effects (25, 26).

With advanced molecular techniques available, further studies on ionizing radiation should elucidate the unanswered genetic questions that Dr. Schull was seeking to resolve.

## Author contributions

The author confirms being the sole contributor of this work and has approved it for publication.

## Footnotes

^1^James F. Crow was one of the leading population geneticists for the twentieth century. He was also mentor to another set of prominent population geneticists including Motoo Kimura (NIG), one of the founders of the Neutral theory of molecular evolution (4).
